# Synergistic effects of fecal microbiota transplantation and andrographolide on gut microbiota modulation in dextran sulfate sodium-induced colitis in mice

**DOI:** 10.3389/fmicb.2026.1762107

**Published:** 2026-03-19

**Authors:** Xuling Luo, Jiajia Feng, Yue Sun, Zhijie Tan, Lan Wang, Yuxiao Bao, Junwan Lu, Jun He, Bin Lu

**Affiliations:** 1 Department of Cell Biology and Genetics, Department of Gastroenterology, School of Basic Medical Sciences and Affiliated Nanhua Hospital, Hengyang Medical School, University of South China, Hengyang, Hunan, China; 2 Department of Hepatobiliary Surgery, The Affiliated Nanhua Hospital, Hengyang Medical School, University of South China, Hengyang, Hunan, China; 3 Department of Ultrasound, The Affiliated Hospital of Yunnan University, Kunming, Yunnan, China; 4 Medical Molecular Biology Laboratory, School of Medicine, Jinhua University of Vocational Technology, Jinhua, Zhejiang, China; 5 National Health Commission Key Laboratory of Birth Defect Research and Prevention, Hunan Provincial Maternal and Child Health Care Hospital, Changsha, Hunan, China

**Keywords:** andrographolide, colitis, fecal microbiota transplantation, gut microbiota, NF-κB

## Abstract

**Background:**

Andrographolide (Andro) and fecal microbiota transplantation (FMT) are emerging treatments for colitis. However, whether their combined administration provides superior efficacy has not been established.

**Methods:**

This study attempted to clarify the reparative effects of FMT, Andro, and their combination on colitis in mice. Research subjects were allocated to: (1) the Control (CTRL) group, (2) the dextran sulfate sodium (DSS) group, (3) the FMT group, (4) the Andro group, and (5) the Andro combined with FMT group. The experiment lasted 15 days, during which weight, colon length, and hematochezia were monitored. Colon tissues were histologically analyzed via HE staining to assess inflammatory infiltration. The concentrations of key serum inflammatory factors were measured using ELISA. WB and IHC were employed to quantify inflammatory factor levels in intestinal tissues. Finally, the taxonomic composition of colonic microbiota was examined with 16S rRNA gene sequencing.

**Results:**

All three treatments mitigated colitis, as indicated by lowered pathological body weight wasting, colon shortening, hematochezia, and inflammation. Serum and intestinal cytokine levels were significantly decreased following treatment. Mechanistic analysis indicated that Andro attenuated inflammatory responses primarily through inhibition of NF-κB. Moreover, 16S rRNA sequencing revealed a beneficial modulation of the gut microbiota by all three treatments compared with the DSS group. Integrated analysis demonstrated that Andro combined with FMT therapy produced superior therapeutic outcomes relative to either intervention alone.

**Conclusion:**

The combined administration of Andro and FMT provides enhanced protection against DSS-induced colitis in mice, highlighting a potential synergistic therapeutic strategy.

## Graphical abstract

**Figure:**
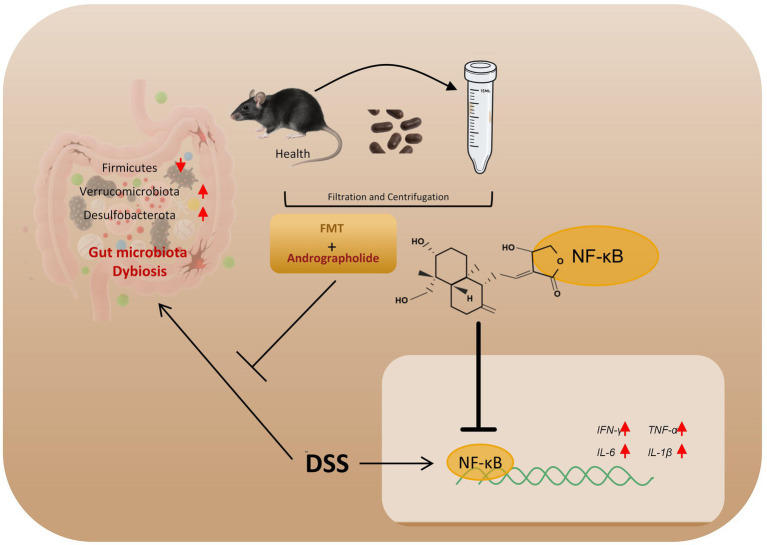

## 1 Introduction

Ulcerative colitis (UC) is a chronic and recurrent intestinal inflammation. Global epidemiological data reveal a persistently rising incidence of UC, with approximately 5 million individuals affected worldwide (Wangchuk et al., 2024). The recurrent disease course not only impairs patients’ quality of life but also increases the risk of colorectal cancer, posing a substantial public health burden globally (Gros and Kaplan, 2023). The pathogenic mechanism of UC involves enteric dysbacteriosis, environmental triggers, and immune dysregulation (Deng et al., 2023). Among these, impairment of the gut microbial ecosystem has been identified as a pivotal driver of mucosal immune imbalance.

FMT has emerged as a therapeutic approach to UC management (Costello et al., 2019; Huang et al., 2024). By transplanting standardized fecal microbiota from healthy donors to patients, FMT can remodel pathogenic microbial communities and restore host-microbial homeostasis (Porcari et al., 2023). However, clinical outcomes remain heterogeneous. Despite all patients receiving microbiota transplantation from the same donor, some still exhibit treatment resistance, indicating that single FMT has limited regulatory capacity over highly activated inflammatory signaling pathways and requires combination with other anti-inflammatory strategies to synergistically suppress inflammatory responses. This suggests that for patients with severe ulcerative colitis, reliance on FMT alone is often insufficient to achieve induction of remission. Therefore, there is an urgent need to develop combination therapies that can simultaneously correct “microbial dysbiosis” and suppress “excessive inflammation” in order to enhance overall treatment efficacy (Li et al., 2016; Costello et al., 2019; Gu et al., 2022; Claytor and Faith, 2025).

Andrographolide (Andro, C_20_H_30_O_5_), the primary bioactive compound in *Andrographis paniculata*, has the effect of heat-clearing and detoxifying (Jadhav and Karuppayil, 2021). This compound exhibits potent inflammation-inhibiting, immunomodulatory, and cytoprotective effects (Xiang et al., 2020; Zeng et al., 2022). Studies have confirmed that Andro can ameliorate inflammatory bowel disease through its remarkable anti-inflammatory, antioxidant, and immunomodulatory effects. In terms of anti-inflammatory activity, this compound inhibits key pro-inflammatory signaling pathways such as nuclear factor-κB (NF-κB) and Mitogen-Activated Protein Kinases (MAPK), thereby effectively reducing the expression of pro-inflammatory cytokines (Kim et al., 2019; Robertson et al., 2025). Regarding antioxidant stress, Andro alleviates intestinal oxidative stress damage and further reduces inflammatory responses via the Nuclear factor erythroid 2-related factor 2 (Nrf2)/Heme oxygenase-1 (HO-1) antioxidant pathway (Shu et al., 2024). Finally, in immunomodulation, Andro can restore immune homeostasis by regulating the balance between Th17 and Treg cells (Zhang et al., 2025). Recent reports have shown that researchers used exosome-like nanoparticles derived from *Paederia scandens* as a carrier to construct an Andro nano-delivery system. This system demonstrated excellent therapeutic effects in experiments, significantly alleviating colitis symptoms and restoring colon length (Peng et al., 2025). Andro alleviates pathological damage in colitis models, although the contribution of gut microbiota to its therapeutic effects remains incompletely understood.

In summary, previous studies have demonstrated that both FMT and andrographolide possess distinct advantages in the treatment of UC, yet each monotherapy has certain limitations. Based on this rationale, we propose a combination therapeutic strategy: utilizing FMT to systematically correct microbial dysbiosis, while employing andrographolide to inhibit key inflammatory pathways such as NF-κB. Moreover, this combination may achieve synergistic inhibition of these two core pathological processes in UC. On one hand, andrographolide, while suppressing inflammation, may create a more favorable microenvironment for the colonization of transplanted microbiota. On the other hand, the healthy microbiota derived from FMT may enhance host anti-inflammatory responses through their metabolites, thereby producing a synergistic anti-inflammatory effect.

Therefore, this study investigates the therapeutic benefits of FMT, Andro, and their combination in acute colitis, meanwhile evaluating their impacts on intestinal microbiota composition. In addition, we explore NF-κB signaling as a potential mechanistic target.

## 2 Materials and methods

### 2.1 Reagents and antibodies

NLRP3 (A5652), IL-6 (A0286), TNF-*α* (A11534) were from Abclonal (Wuhan, China). NF-κB (8242S), IL-1β (12242S) were from CST (Beverly, USA). GAPDH (60004-1-Ig) was from Proteintech (Wuhan, China). DSS (MB5535) was obtained from Meilunbio (Dalian, China). Andro (S2261) was obtained from Selleck (Houston, USA).

### 2.2 Animals and study design

**Figure 1:**
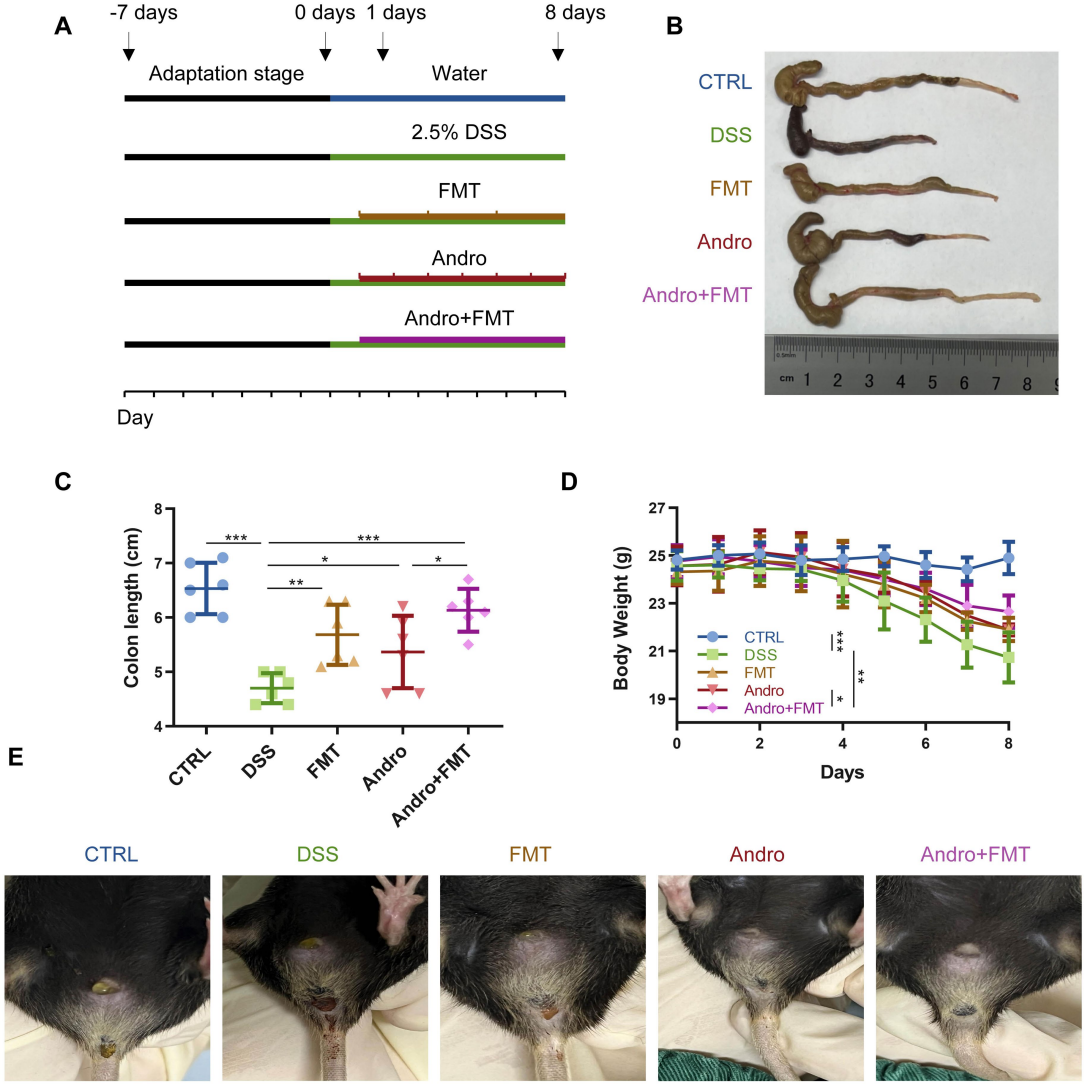
Alleviation of colitis by combined Andro and FMT treatment. **(A)** Experimental design (*n* = 6). **(B)** Colon photographs. **(C)** Final colon length. **(D)** Comparison of body weight changes **(E)** The situation of bloody stool. *p < 0.05; **p < 0.01; ***p < 0.001.

Animal research was performed under the protocols approved by the Ethics Committee of the University of South China (0087). Male C57BL/6 J mice (6–8 weeks old) were purchased from GemPharmatech (Nanjing, China). To minimize environmental stress, mice were acclimated for one week prior to experimentation under specific pathogen-free (SPF) conditions. The study timeline is outlined in Figure 1A. Mice were assigned to: (1) Control (CTRL) (regular drinking water), (2) DSS, (3) FMT (200 μL healthy microbiota suspension via oral gavage per administration), (4) Andro (50 mg/kg/day Andro via oral gavage), and (5) Andro combined with FMT group. Mice in the DSS, FMT, Andro, and Andro combined with FMT groups received 2.5% DSS solution for 8 days. Body weight was recorded daily, with clinical assessments for diarrhea and fecal bleeding. On day 15, after a 12-h fasting period, final body weights were documented. Mice were then euthanized by cervical dislocation following retro-orbital venous plexus puncture under anesthesia for blood collection. Colon tissues were immediately excised for subsequent analyses: Colon length was measured. Segments for histology were fixed in 4% paraformaldehyde. The left colon tissues and luminal contents were stored at −80 °C.

### 2.3 Preparation of stool sample

Fresh fecal samples from healthy mice were resuspended in sodium chloride solution (0.9%) at a ratio of 200 mg feces per 2 mL. The mixture was homogenized until no visible particulate matter remained, followed by filtration through a sterile mesh filter to remove undigested food residues and large particles. The filtrate was collected in sterile centrifuge tubes, vigorously vortexed for 5 min, and then centrifuged. The supernatant was combined with 10% sterile glycerol and cryopreserved at −80 °C.

### 2.4 Hematoxylin–eosin (HE) staining

Colon tissues from each group were subjected to histopathological examination by hematoxylin and eosin (HE) staining after standard processing (fixation, dehydration, embedding, and sectioning).

### 2.5 Immunohistochemistry (IHC) staining

Following deparaffinization, rehydration, epitope retrieval, inactivation of endogenous peroxidases, and non-specific binding blockade, histological sections were incubated with primary and secondary antibodies. After TBST washes, immunohistochemical signals were visualized using DAB, counterstained with Mayer’s hematoxylin (Solarbio, Beijing, China), and examined microscopically.

### 2.6 Enzyme-linked immunosorbent assay

The serum concentrations of IL-1β, IFN-*γ*, TNF-*α*, and IL-6 were assessed via enzyme-linked immunosorbent assay using kits from Jiangsu Meimian Industrial (Jiangsu, China).

### 2.7 Western blot analysis

After homogenization and centrifugation of colorectal tissues, protein concentration was measured with a BCA assay kit (Pierce, Rockford, USA). Samples were then heated with loading buffer, resolved by electrophoresis, and transferred to a membrane for blocking and incubation with primary and secondary antibodies. After thorough TBST washing, signals were visualized with an ECL kit (Thermo Fisher Scientific, Waltham, USA). Quantitative analysis was then performed using ImageJ software.

### 2.8 Molecular docking

The interaction mode between human NF-κB and andrographolide was systematically predicted using integrated computational biology approaches. First, the amino acid sequence of human NF-κB (accession number: P19838) was retrieved from the UniProt database.^1^ Subsequently, the three-dimensional structure of NF-κB and its complex with andrographolide were modeled using AlphaFold3.^2^ To evaluate binding affinity, semi-flexible molecular docking was performed using AutoDock, with the docking box centered on the active site region predicted by AlphaFold3, and the binding free energy was calculated. Finally, the docked complex was visualized and analyzed using PyMOL.

### 2.9 Cellular thermal shift assay (CETSA)

Approximately 1 × 10^7^ 293 T underwent five freeze–thaw cycles. Following centrifugation, the supernatants were divided into two aliquots. These lysates were treated with either Andro (30 μg/mL) or DMSO vehicle control for 120 min. Subsequently, both Andro- and DMSO-treated lysates were equally partitioned into six samples and subjected to a thermal gradient (37–52 °C) for 10 min. After cooling to room temperature, soluble proteins were harvested by centrifugation at 15,000 g for 40 min and analyzed via Western blotting using antibodies specific to target proteins.

### 2.10 Microbial community analysis

Genomic DNA was extraction from fecal samples in each group. Nucleic acid concentration was measured with a microplate reader, and its quality was assessed through PCR amplification and electrophoretic detection. Subsequently, multiple primers targeting different microbial types were designed. Libraries were constructed following steps such as PCR amplification and purification. Finally, the prepared libraries were sequenced.

### 2.11 Statistical analysis

Statistical analyses were performed using GraphPad Prism (GraphPad Software, San Diego, CA, USA). Intergroup comparisons were conducted with unpaired Student’s *t*-test. Microbial abundance profiles in colonic tissues were visualized using OriginPro (OriginLab Corporation, Northampton, USA). Quantitative data are expressed as mean ± standard deviation (SD). A two-tailed *p* value < 0.05 was considered statistically significant.

## 3 Results

### 3.1 Andro combined with FMT alleviates colitis in mice

To study the efficacy of FMT, Andro, and their combination on colitis, mice were induced to develop colitis via administration of 2.5% DSS. Mice were then treated with Andro for seven consecutive days (days 1–7), FMT for 3-day (on days 1, 3, and 5), or a combination of both (Figure 1A). Body weight, colon length, and bloody stool were assessed (Figures 1B–E). As evidenced in Figures 1B,C, in comparison to the DSS group, both Andro and FMT treatment inhibited DSS-induced colon shortening, with the combination therapy further alleviating this effect. DSS-treated mice exhibited typical colitis symptoms including weight loss and bloody stools. Both Andro and FMT ameliorated these symptoms to some extent, while the combination therapy demonstrated the most significant improvement (Figures 1D,E).

### 3.2 Andro combined with FMT reduces DSS-induced colonic inflammation

**Figure 2:**
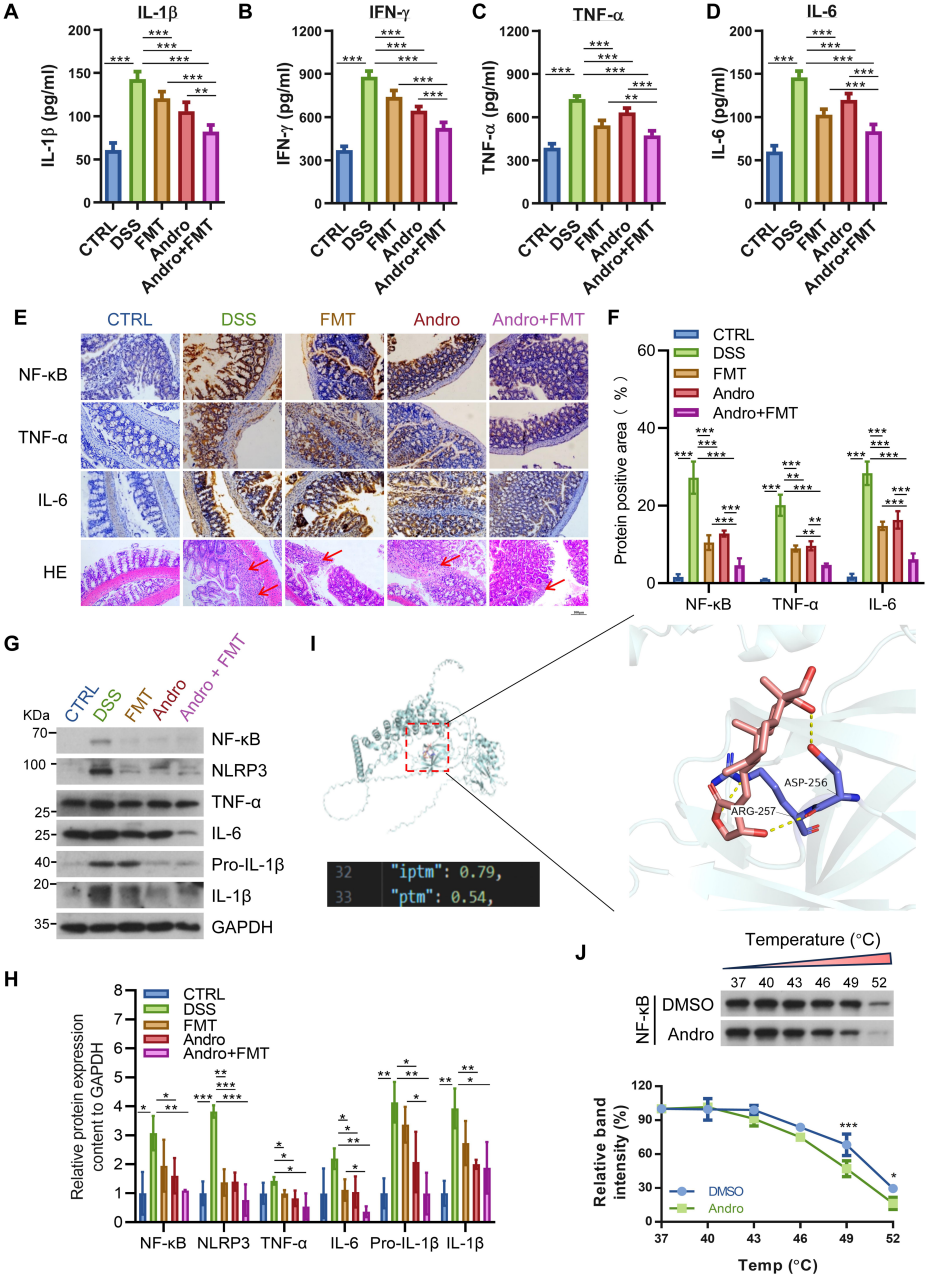
Andro combined with FMT reduces colonic inflammation by NF-κB. The levels of **(A)** IL-1β, **(B)** IFN-*γ*, **(C)** TNF-*α*, and **(D)** IL-6 (pg/ml) in the serum (*n* = 6). **(E)** Representative images of NF-κB, TNF-*α*, IL-6, and HE staining of mouse colon. Scale bar is 100 μm. **(F)** Quantitative analysis of the positive area percentage for NF-κB, TNF-*α*, and IL-6 from the immunohistochemical images shown in (E) (*n* = 3). **(G)** Expression of NF-κB, NLRP3, TNF-*α*, IL-6, and IL-1β proteins in intestinal tissue of different groups. **(H)** Were normalized to GAPDH and expressed as fold change relative to the control group (*n* = 3) **(I)** Interaction simulations of Andro with NF-κB. **(J)** CETSA (293 T cells, at 37, 40, 43, 46, 49, and 52 °C). *p < 0.05; **p < 0.01; ***p < 0.001.

ELISA analysis revealed elevated serum cytokines levels in DSS-treated mice, which were reversed by FMT, Andro, or combination therapy (Figures 2A–D). Immunohistochemistry (Figures 2E,F) and Western blot (Figures 2G,H) confirmed reduced intestinal inflammatory mediators. HE staining also revealed that treated with FMT, Andro, or Andro combined with FMT exhibited reduced inflammation intensity compared to the DSS group, with the combined therapy achieving significantly greater efficacy than individual treatments (Figure 2E). Molecular docking simulations preliminarily revealed that Andro potentially binds directly to the target protein NF-κB *in vitro*, with specific binding sites at residues Asp-256 and Arg-257. The calculated binding energy was −7.7 kcal/mol, indicating a strong binding affinity (Figure 2I). Cellular thermal shift assay (CETSA) further demonstrated the Andro-NF-κB interaction. Andro treatment significantly reduced the thermal stability of NF-κB (Figure 2J), consistent with accelerated denaturation upon ligand binding. This supports a model wherein Andro directly engages NF-κB, potentially inducing conformational changes that destabilize the protein. Together with preceding data, these results establish Andro as a specific cellular binder of NF-κB. In summary, Andro exerts direct anti-inflammatory effects by targeting and binding to NF-κB, while its combination with FMT synergistically enhances the alleviation of DSS-induced colonic inflammation.

### 3.3 Impact on microbial abundance at phylum level

**Figure 3:**
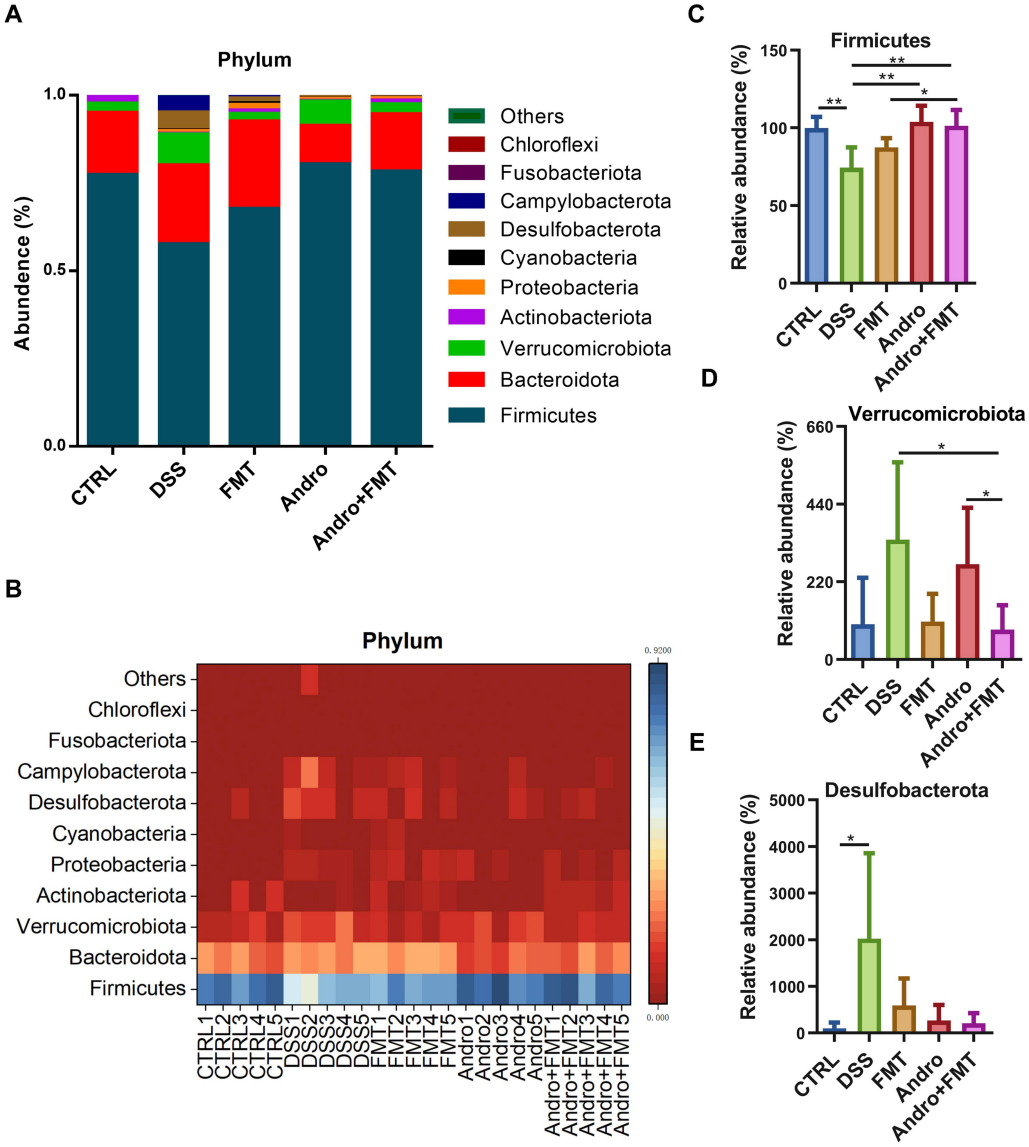
Phylum-level microbial composition in response to different treatments. **(A, B)** Ten most abundant microorganisms by phylum—level percentage (*n* = 5). **(C)** Firmicutes, **(D)** Verrucomicrobiota, **(E)** Desulfobacterota. *p < 0.05, **p < 0.01.

The top ten microbial phyla are shown in Figures 3A,B. Firmicutes, Bacteroidota, and Verrucomicrobiota accounted for the majority of the community. The proportions of Firmicutes in the CTRL, DSS, FMT, Andro, and Andro combined with FMT groups were 77.22, 57.53, 67.61, 80.24, and 78.23%, respectively. Bacteroidota accounted for 17.75, 22.46, 24.89, 10.97, and 16.35% in these groups, while Verrucomicrobiota constituted 2.59, 8.79, 2.18, 7.00, and 2.79%, respectively. The DSS group showed a pronounced reduction in Firmicutes abundance (Figure 3C). In contrast, all three treatment groups, FMT, Andro, and combination therapy, showed increased Firmicutes levels, with the greatest enrichment observed in the Andro and combination groups. Furthermore, Verrucomicrobiota abundance in the combination group was lower compared to that in the DSS group (Figure 3D). DSS treatment also led to an elevation in Desulfobacterota (Figure 3E). Despite the lack of significant differences in Desulfobacterota levels among all three treatments, a consistent downward trend was noted in these treatment groups compared to the DSS group.

### 3.4 Impact on microbial abundance at class level

**Figure 4:**
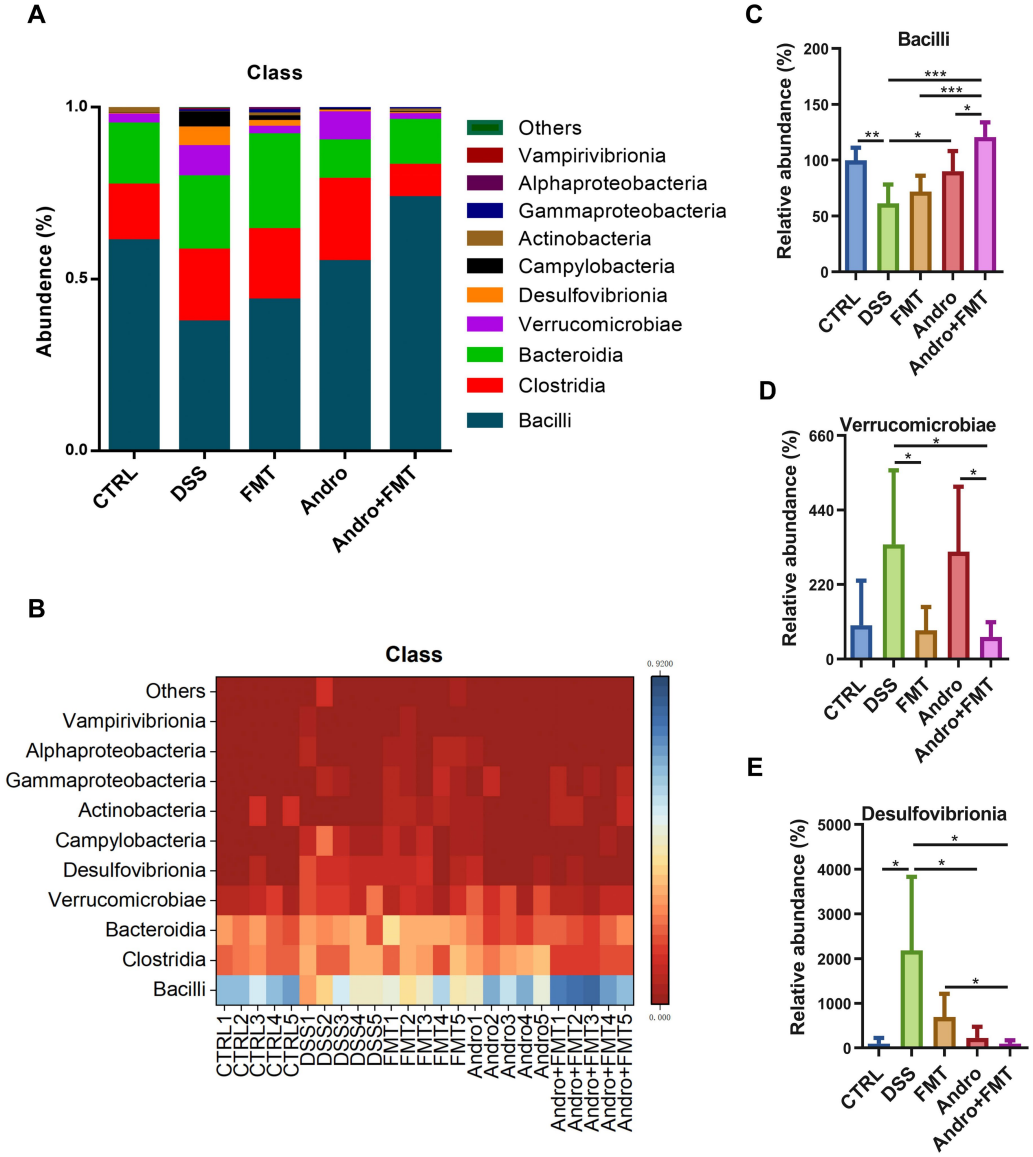
Class-level microbial composition in response to different treatments. **(A, B)** Top ten microorganisms in the class-level (*n* = 5), **(C)** *Bacilli*, **(D)** *Verrucomicrobiae*, **(E)** *Desulfovibrionia*. *p < 0.05, **p < 0.01, ***p < 0.001.

Figures 4A,B display the top ten microorganisms at the class level across experimental groups. The three most dominant microorganisms and their relative abundances in each group were as follows: CTRL group: Bacilli (60.95%), Clostridia (16.26%), and Bacteroidia (17.75%); DSS group: Bacilli (37.35%), Clostridia (20.85%), and Bacteroidia (21.41%); FMT group: Bacilli (43.76%), Clostridia (20.37%), and Bacteroidia (27.65%); Andro group: Bacilli (54.88%), Clostridia (23.99%), and Bacteroidia (11.10%); Andro combined with FMT group: Bacilli (73.55%), Clostridia (9.3%), and Bacteroidia (13.18%). As shown in Figure 4C, compared to the DSS group, the abundance of Bacilli in the colon was significantly increased in the CTRL, Andro, and Andro combined with FMT groups while the FMT group showed no statistically significant increase. In Figure 4D, the abundance of Verrucomicrobiae was significantly reduced in the FMT and Andro combined with FMT groups compared to the DSS group. Figure 4E demonstrates that the abundance of Desulfovibrionia in the colon was significantly lower in the CTRL, Andro, and Andro combined with FMT groups relative to the DSS group. Although the FMT group exhibited no statistically significant reduction, a downward trend in Desulfovibrionia levels was still observed compared to the DSS group.

### 3.5 Impact on microbial abundance at order level

**Figure 5:**
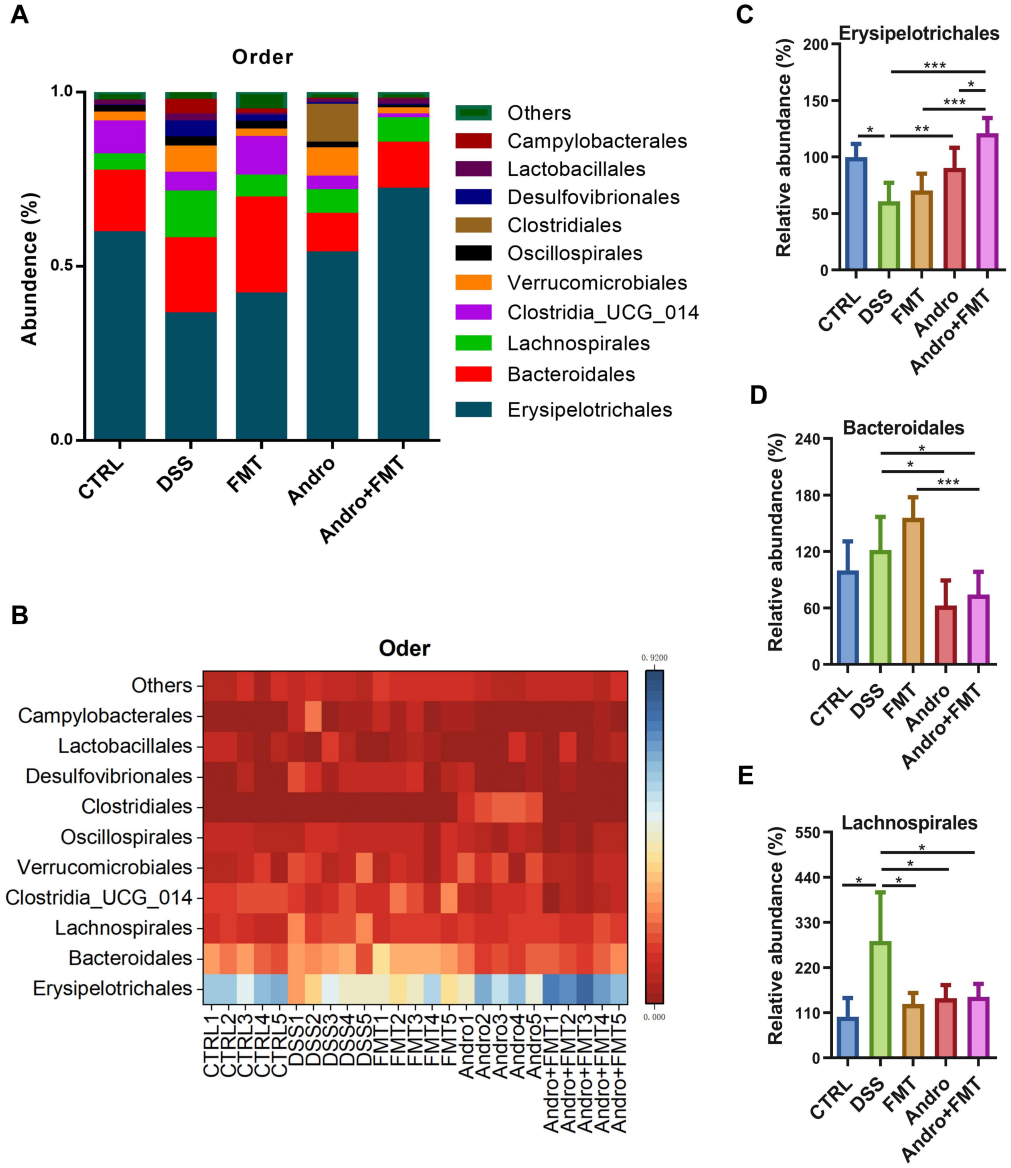
Order-level microbial composition in response to different treatments. **(A, B)** Top ten microorganisms with the percentage of order-level (*n* = 5), **(C)** *Erysipelotrichales*, **(D)** *Bacteroidales*, **(E)** *Lachnospirales*. *p < 0.05, **p < 0.01, ***p < 0.001.

Figures 5A,B depict the top ten microorganisms at the order level and their relative abundances across experimental groups. The three most dominant orders and their proportions in each group were as follows: CTRL group: Erysipelotrichales (59.40%), Bacteroidales (17.74%), and Lachnospirales (4.70%); DSS group: Erysipelotrichales (36.18%), Bacteroidales (21.58%), and Lachnospirales (13.39%); FMT group: Erysipelotrichales (41.83%), Bacteroidales (27.65%), and Clostridia_UCG-014 (11.10%); Andro group: Erysipelotrichales (53.62%), Bacteroidales (11.10%), and Lachnospirales (6.82%); Andro combined with FMT group: Erysipelotrichales (71.96%), Bacteroidales (13.16%), and Lachnospirales (6.99%). As shown in Figure 5C, compared to the DSS group, the abundance of Erysipelotrichales in the colon was significantly increased in the Andro and Andro combined with FMT groups, while the FMT group showed no statistically significant increase, though an upward trend was observed. Both the Andro and Andro combined with FMT groups exhibited a significant reduction in Bacteroidales levels. Notably, Figure 5D reveals that the abundance of Bacteroidales in the FMT group displayed a tendency to increase rather than decrease compared to the DSS group, although no statistically significant differences were observed between groups. In Figure 5E, compared to the DSS group, the abundance of Lachnospirales in the colon showed a downward trend in the CTRL, FMT, Andro, and Andro combined with FMT groups; moreover, these changes were statistically significant.

### 3.6 Impact on microbial abundance at family level

**Figure 6:**
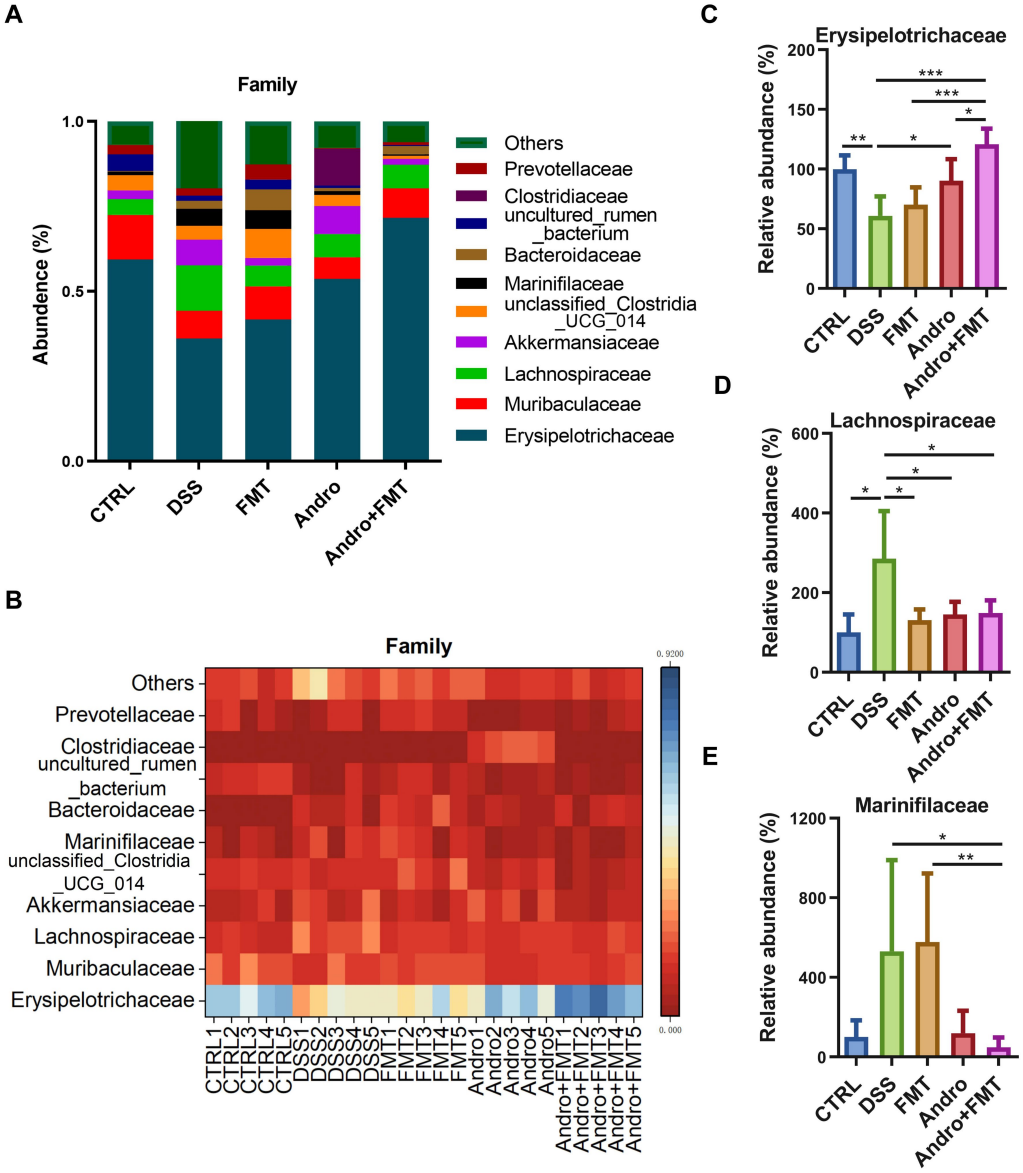
Family-level microbial composition in response to different treatments. **(A, B)** Ten most abundant microorganisms by family-level percentage (*n* = 5). **(C)** *Erysipelotrichaceae*, **(D)** *Lachnospiraceae*, **(E)** *Marinifilaceae*. *p < 0.05, **p < 0.01, ***p < 0.001.

Figures 6A,B depict the top ten microorganisms ranked by relative abundance at the family level. The dominant microorganisms and their proportions in the CTRL, DSS, and Andro combined with FMT groups were Erysipelotrichaceae (59.38, 36.11, and 71.70%), Muribaculaceae (13.06, 8.19, and 8.62%), and Lachnospiraceae (4.68, 13.36, and 6.98%), respectively. In the FMT group, the most abundant microorganisms were Erysipelotrichaceae (41.70%), Muribaculaceae (9.76%), and unclassified_Clostridia_UCG_014 (8.57%), while the Andro group exhibited dominance of Erysipelotrichaceae (53.59%), Clostridiaceae (10.77%), and Akkermansiaceae (8.21%). The Andro and Andro combined with FMT groups displayed a notable elevation in Erysipelotrichaceae abundance compared to the DSS group. In contrast, the increase observed in the FMT group was not statistically significant (Figure 6C). Figure 6D demonstrates that the abundance of Lachnospiraceae was reduced in FMT, Andro, and Andro combined with FMT groups relative to DSS group. A decrease in Marinifilaceae was uniquely observed in the Andro combined with FMT groups (Figure 6E).

### 3.7 Effects on *α*-diversity of colonic microbiota in mice

**Figure 7:**
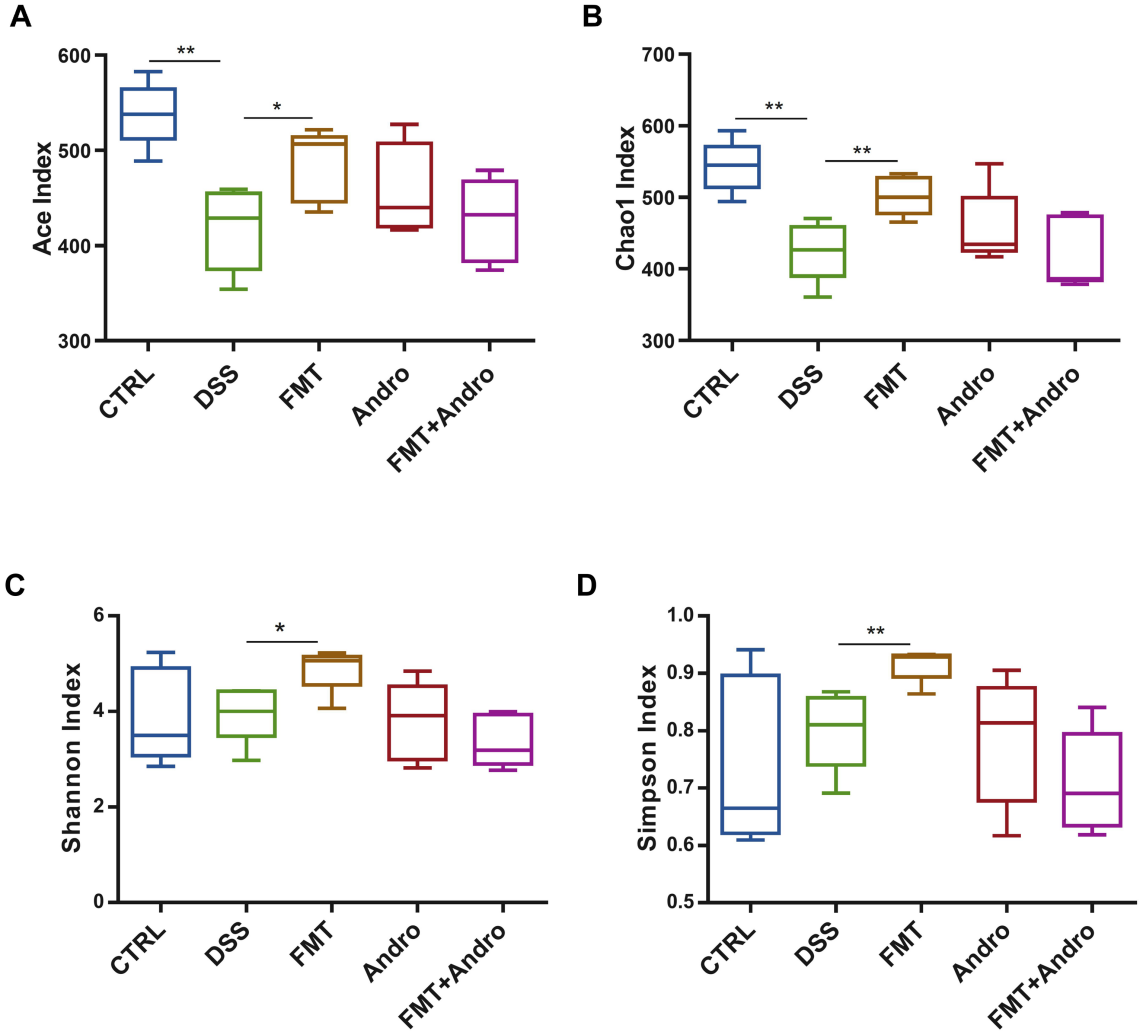
*α*-Diversity of colonic microbiota: **(A)** Ace index, **(B)** Chao1 index, **(C)** Shannon index, and **(D)** Simpson index. *p < 0.05, **p < 0.01.

Assessment of 16S rRNA sequencing data from colonic content specimens (Figures 7A–D) revealed the following: The FMT group exhibited significantly enhanced microbial richness (Ace and Chao1 indices) and diversity (Shannon and Simpson indices) versus the DSS group. The CTRL group exhibited greater Ace and Chao1 indices than the DSS group, though no notable disparities were identified for Shannon or Simpson indices. While the Andro group and Andro combined with FMT group displayed no statistically significant variations in the four *α*-diversity indices, both groups demonstrated upward trends in microbial richness. In summary, the FMT group most effectively restored intestinal microbial diversity in mice following DSS-induced dysbiosis.

### 3.8 Beta-diversity and LEfSe analysis of gut microbiota

**Figure 8:**
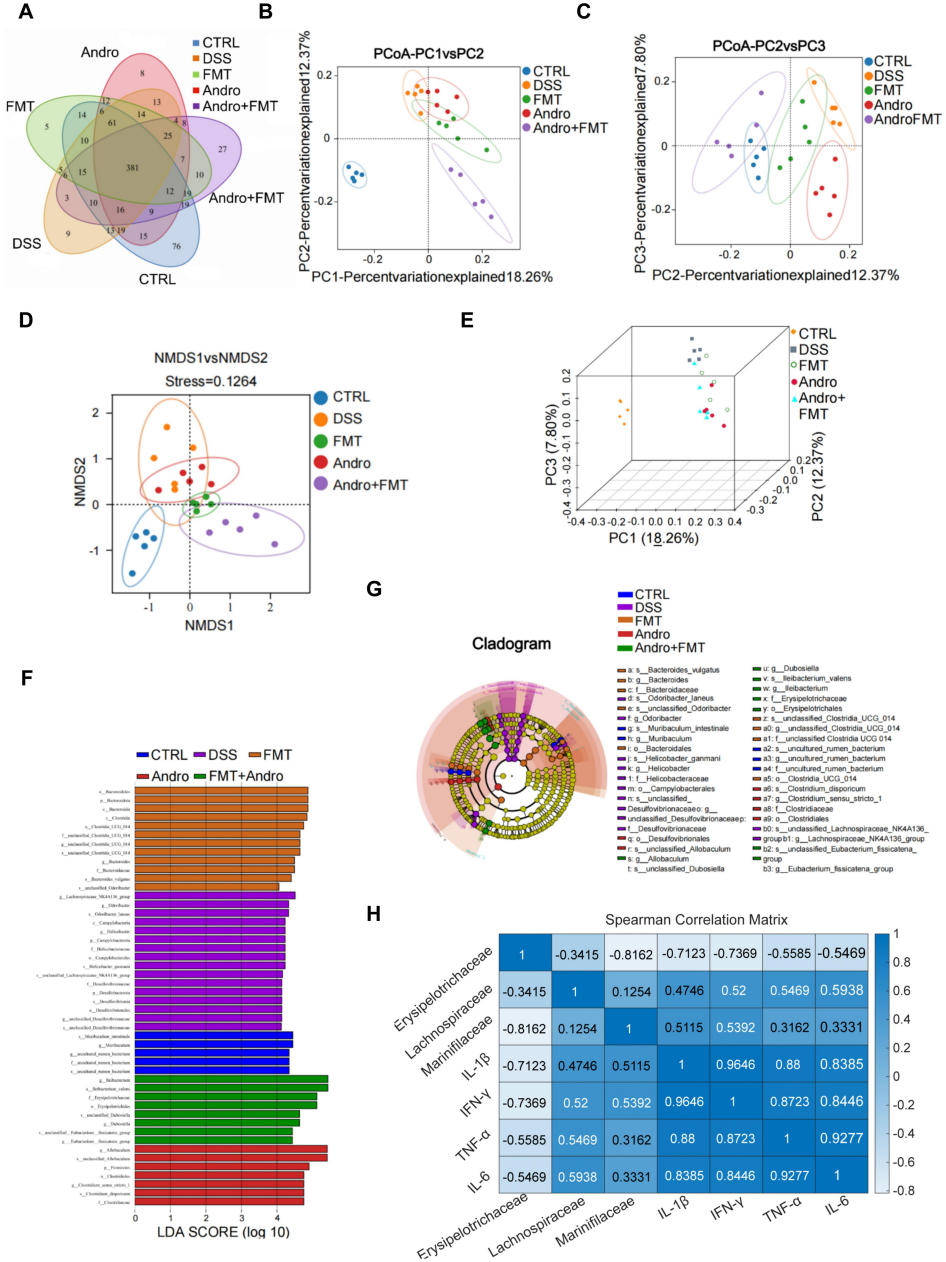
Beta-diversity and LEfSe analysis. **(A)** Venn, **(B)** PCoA-PC1 vs. PC2, **(C)** PCoA-PC2 vs. PC3, **(D)** NMDS, **(E)** PLS-DA 3D, **(F, G)** LEfSe. **(H)** Spearman correlation analysis between key microbial families and inflammatory parameters.

In Figures 8A, a Venn diagram reveals the unique and shared OTU across groups. CTRL, DSS, FMT, Andro, and Andro combined with FMT groups contained 76, 9, 5, 8, and 27 unique OTU, respectively, while 381 OTU were shared among all five groups, indicating distinct differences in microbial composition. Beta-diversity analysis was conducted to assess overall community structure (Figures 8B–E). Principal coordinates analysis (PCoA) (Figures 8B,C) showed that the first three principal components accounted for 18.26, 12.37, and 7.80% of the variance, respectively. DSS treatment altered colonic microbial community structure. Notably, FMT, Andro, and Andro combined with FMT groups exhibited distinct clustering patterns compared to DSS group, suggesting these interventions ameliorated DSS-induced microbial dysbiosis. NMDS (Figure 8D), and PLS-DA 3D (Figure 8E) yielded consistent conclusions, further validating the structural divergence between DSS and treatment groups. LEfSe analysis identified key discriminative microbial taxa across groups (Figures 8F,G): The CTRL group: Dominant biomarkers included Muribaculum and unclassified Bacterium. The DSS group: Enriched taxa comprised Lachnospiraceae, Odoribacter, Campylobacteria, and Helicobacter. The FMT group: Key biomarkers were Bacteroidota, Clostridia, and Odoribacter. The Andro group: Discriminative taxa included Allobaculum, Firmicutes, and Clostridium. The Andro combined with FMT group: Biomarkers were Ileibacterium, Erysipelotrichaceae, and Dubosiella. To explore the relationship between gut microbes and serum inflammatory factors in mouse colitis, we investigated the correlation between microbes in the mouse colon and various inflammatory factors (Figure 8H).

## 4 Discussion

UC, a therapeutically challenging disease in modern clinical practice, exhibits a persistently rising global incidence. Although therapeutic options for UC continue to expand, drug efficacy remains suboptimal with clinical remission rates not exceeding 60% (Le Berre et al., 2023). Consequently, a subset of patients still require proctocolectomy due to pharmacologic refractoriness, posing severe threats to human health. UC pathogenesis involves genetically susceptible individuals developing the disease following environmental triggers, with intestinal dysbiosis recognized as a core driver of mucosal immune imbalance. FMT, initially employed for Clostridioides difficile infection where it demonstrates superiority over standard therapies (fidaxomicin and vancomycin), promotes donor-derived strain colonization (primarily Lactobacillales) in recipient intestines through the stimulator of interferon genes (STING) pathway, thereby remodeling the gut microbiota and ameliorating colitis (Pu et al., 2024; Dai et al., 2025). During the preceding decade, growing recognition has established the gut microbiome as both an integral component of host physiology and a crucial therapeutic target (Fu et al., 2025). However, clinical translation faces significant challenges, such as donor-recipient compatibility issues leading to heterogeneous responses, safety concerns in immunocompromised patients (pathogen transmission risk), and lack of standardized protocols for microbiota preparation and administration. These limitations necessitate combinatorial approaches for moderate-to-severe UC (Claytor and Faith, 2025).

Andro, the primary bioactive constituent of *Andrographis paniculata*, serves as the fundamental basis for its pharmacological efficacy. It exhibits therapeutic benefits such as anti-inflammatory and antioxidant properties (Wang et al., 2018; Xiang et al., 2020). Previous studies demonstrate that Andro not only ameliorates acute colitis in mice by inhibiting inflammatory responses through AMP-activated protein kinase pathway activation but also mitigates oxidative stress, repairs the intestinal mucosal barrier, and alleviates UC symptoms via the Nrf2/HO-1 pathway (Kim et al., 2019; Shu et al., 2024). Whether combined administration of FMT and Andro enhances UC remission remained unclear. This study confirms that both Andro and FMT individually alleviate colitis symptoms. Crucially, combination therapy (Andro combined with FMT) demonstrates superior efficacy compared to monotherapies.

Inflammatory cytokines serve as key biomarkers for assessing inflammation severity. Research indicates increased expression of IL-1β, IL-6, and TNF-*α* during intestinal inflammation (Tan et al., 2023). NF-κB, the master transcriptional regulator of inflammatory responses, promotes transcription of numerous inflammation-related genes (Zhong et al., 2016; Wang, Y. et al., 2024; Sun et al., 2025). Notably, IL-1β also can reactivate NF-κB through receptor binding and amplify inflammatory signaling (Shen et al., 2022). This study found that the combination therapy not only significantly reduced local intestinal inflammation but also effectively decreased the concentration of inflammatory factors in the systemic circulation.

Andro shows anti-inflammatory effects in multiple diseases, primarily attributed to NF-κB inhibition (Yang et al., 2023). To investigate potential binding interactions, molecular docking analysis was performed, revealing that Andro likely binds directly to ASP-256 and ARG-257 residues on the NF-κB protein. Docking models further confirmed structural compatibility between the complex and native protein conformations, with accurate spatial prediction of subunit arrangements. Additionally, CETSA demonstrated accelerated thermal denaturation of NF-κB upon Andro treatment. Although ligand binding typically stabilizes target proteins against thermal aggregation, the observed reduction in NF-κB stability here suggests that Andro may induce conformational changes, thereby facilitating denaturation under heat stress. This counterintuitive phenomenon, in which compound binding reduces thermal stability, has been documented and aligns with our findings (Wu et al., 2023). This indicates that direct compound binding reduces NF-κB stability. Complementary studies confirm that FMT effectively reduces NF-κB expression levels. Published evidence suggests that Andro inhibits NF-κB activity at multiple levels, including nuclear translocation, DNA binding, and post-translational modification (Bao et al., 2009; Hsieh et al., 2011). However, the specific mechanism by which Andro inhibits NF-κB in the DSS-induced colitis model requires further investigation. These findings suggest synergistic suppression of NF-κB by Andro combined with FMT through dual mechanisms: (1) Andro directly inhibits NF-κB-driven cytokine production, creating an anti-inflammatory microenvironment conducive to donor microbiota colonization; (2) reciprocally, FMT-derived beneficial bacteria enhance intestinal barrier integrity and amplify Andro’s immunomodulatory effects, forming a positive feedback loop. Collectively, as the central hub of inflammation regulation, NF-κB is synergistically suppressed by Andro and FMT to ameliorate colitis. To further validate the specificity of NF-κB pathway inhibition by the combined Andro and FMT therapy and to exclude potential interference from other signaling pathways, future studies will employ intestinal epithelial cell-specific NF-κB (p65) deficient conditional knockout mice for *in vivo* experiments. This approach will elucidate the specific regulatory role of the NF-κB pathway in mediating the intestinal mucosal protection and anti-inflammatory effects of the combination therapy, thereby confirming the specificity of NF-κB targeting by the combined treatment and further enhancing the rigor and reliability of the mechanistic findings in this study.

Gut microorganisms constitute pivotal factors in host metabolism and represent a promising novel therapeutic approach. The immense gut microbiota engages in dynamic bidirectional cross-talk with the host: while the host affords habitat and nutrients, commensal microbes modulate multiple physiological processes including immune homeostasis, energy metabolism, and neurocognition (Zaiss et al., 2021; Wang, Z. et al., 2024). Additionally, patients with enteritis develop dysbiosis, which is typically characterized by phylum-level imbalance healthy adults, Firmicutes and Bacteroidota constitute over 90% of gut microbiota (Qin et al., 2010). This study reveals that healthy murine gut microbiota primarily comprises five phyla: Firmicutes (77.2%), Bacteroidota (17.7%), Verrucomicrobiota (2.6%), Actinobacteriota (1.9%), and Proteobacteria (0.1%), showing partial compositional similarity to humans. The phylum Firmicutes encompasses numerous important short-chain fatty acid (SCFA)-producing bacteria, which play roles in anti-inflammation, preservation of intestinal barrier intactness, and immune regulation. For example, *Clostridium sporogenes*, a representative strain of Firmicutes, demonstrates remarkable protective effects in DSS-induced colitis models through the production of diverse metabolites, including indole-3-propionic acid, SCFAs, and branched-chain fatty acids (Wu et al., 2020). Prior literature has reported that the abundances of Verrucomicrobiota and Desulfobacterota in the DSS model are significantly increased, indicating that DSS treatment may promote the proliferation of Verrucomicrobia and Desulfobacterota. The mechanism may involve the alterations in the intestinal environment after DSS disrupts the intestinal barrier (Hua et al., 2021; Wu et al., 2021; Ren et al., 2022). In the current investigation, relative to the CTRL group, the DSS treatment substantially lowered the abundances of Firmicutes, while the abundances of Verrucomicrobiota and Desulfobacterota were elevated. In comparison with the DSS treatment, FMT treatment demonstrated a partial reversal of the altered abundances for these bacterial phyla, though this did not reach statistical significance. In contrast, Andro treatment significantly increased the proportion of Firmicutes in colitis model. Notably, Andro combined with FMT treatment remarkably reverses both Firmicutes and Verrucomicrobiota abundances relative to the DSS group.

To further elucidate the specific microbial shifts at a finer taxonomic resolution, we analyzed the family-level compositional changes. Erysipelotrichaceae belongs to the phylum Firmicutes and is closely associated with host lipid metabolism and inflammatory responses (Kaakoush, 2015). Studies have reported that Erysipelotrichaceae participates in secondary bile acid production by encoding the *bsh* gene; reduced abundance of this gene directly leads to disturbances in intestinal bile acid metabolism, thereby affecting bile acid receptor signaling and ultimately contributing to the pathogenesis of intestinal inflammation (Labbé et al., 2014). These findings suggest that Erysipelotrichaceae represents a core bacterial cluster negatively correlated with UC. In the present study, the abundance of Erysipelotrichaceae was significantly reduced in DSS-induced colitis compared to the control group. Moreover, Erysipelotrichaceae abundance showed significant negative correlations with all pro-inflammatory cytokines tested, particularly IL-1β (*r* = −0.71) and IFN-*γ* (*r* = −0.74), supporting its role as a beneficial taxon whose restoration contributes to the anti-inflammatory efficacy. Notably, treatment with Andro alone, and particularly the combination therapy, significantly restored its abundance. Lachnospiraceae, another Firmicutes family well recognized for its butyrate-producing capacity (Vacca et al., 2020), exhibited a marked increase in the DSS group. Previous research has demonstrated that in *TLR5*-deficient mice transiently colonized with adherent-invasive *Escherichia coli* (AIEC), chronic intestinal inflammation leads to a sustained increase in Lachnospiraceae abundance, which persists even after AIEC is cleared by the host. This family has been identified as a microbial source of flagellins isolated from patients with Crohn’s disease, and flagellins are important pro-inflammatory microbial components that trigger intestinal inflammation (Jellbauer and Raffatellu, 2014; Vacca et al., 2020). Marinifilaceae belongs to the phylum Bacteroidota. Studies have reported that TLR2-deficiency leads to a significant increase in the abundance of Marinifilaceae and other bacterial families in the mouse gut. Moreover, the relative abundance of Marinifilaceae shows a highly significant negative correlation with the expression of genes involved in cell cycle signaling pathways, suggesting that TLR2 exacerbates DSS-induced intestinal injury through a Marinifilaceae-dependent attenuation of cell cycle signaling (Shi et al., 2024). Additional research has shown that the relative abundance of Marinifilaceae is closely associated with DSS-induced inflammatory responses, with its abundance changes significantly positively correlated with colonic tissue levels of pro-inflammatory cytokines TNF-*α*, IL-1β, and IL-6 (Zhai et al., 2019). In the present study, the combination therapy significantly reduced the abundance of Marinifilaceae. Marinifilaceae abundance was positively correlated with IL-1β and IFN-γ levels, consistent with its reported pro-inflammatory potential. Collectively, these findings suggest that andrographolide and FMT play a critical role in reversing colitis-associated dysbiosis, with the combination therapy achieving more pronounced restorative effects.

In our DSS colitis model, although FMT monotherapy significantly increased *α*-diversity indices (Ace, Chao1, Shannon, Simpson), while Andro alone showed a marginal improving trend. Paradoxically, the combined intervention exhibited lower *α*-diversity increment than that in the single FMT group. This apparent contradiction may be rationalized by *β*-diversity and LEfSe analyses. The combined treatment group showed the microbiota structure closest to the healthy state in PCoA, NMDS, and PLS-DA. LEfSe identified specific enrichment of key functional bacteria such as Dubosiella and Ileibacterium, suggesting that Andro may selectively suppress opportunistic pathogens while optimizing the niche for FMT-derived core functional bacteria. Thus, the combination shifts microbial restoration from “quantitative expansion” to “functional reconstruction.” Although the species richness (*α*-diversity) increased less markedly with combination therapy, it significantly enhanced the anti-inflammatory metabolic functions of the microbiota and intestinal barrier repair capacity through enriching immunomodulatory (Dubosiella) and mucin-degrading bacteria (*Eubacterium fissicatena* group), ultimately exceeding monotherapy outcomes (Li et al., 2022, 2024).

## 5 Conclusion

This study confirms that both Andro and FMT monotherapies alleviate DSS-induced colitis in mice, while their combination (Andro combined with FMT) exhibits marked synergistic efficacy (Graphical abstract). This synergy manifests not only in superior mitigation of weight loss, colon shortening, and histopathological damage but also in stronger suppression of systemic and intestinal pro-inflammatory cytokines. Critically, 16S rRNA sequencing analysis reveals that combination therapy surpasses single interventions in restoring gut microbiota composition, indicating that its mechanism likely involves regulating microbiota-immunity interplay, with NF-κB suppression as the linchpin mechanism. These results offer compelling evidence supporting the clinical translation of phytochemical-microbiota integrated therapies for UC management.

## Data Availability

The data presented in this study are publicly available. The data can be found at: https://www.ncbi.nlm.nih.gov/sra, accession PRJNA1356674.

## Footnotes

1
https://www.uniprot.org/

2
https://www.alphafoldserver.com
